# Supplementary figures and images for: Bridging of nucleosome-proximal DNA double-strand breaks by PARP2 enhances its interaction with HPF1
Source: PLoS One. 2020 Nov 3;15(11):e0240932. doi: 10.1371/journal.pone.0240932 (PMC7608914; doi:10.1371/journal.pone.0240932)

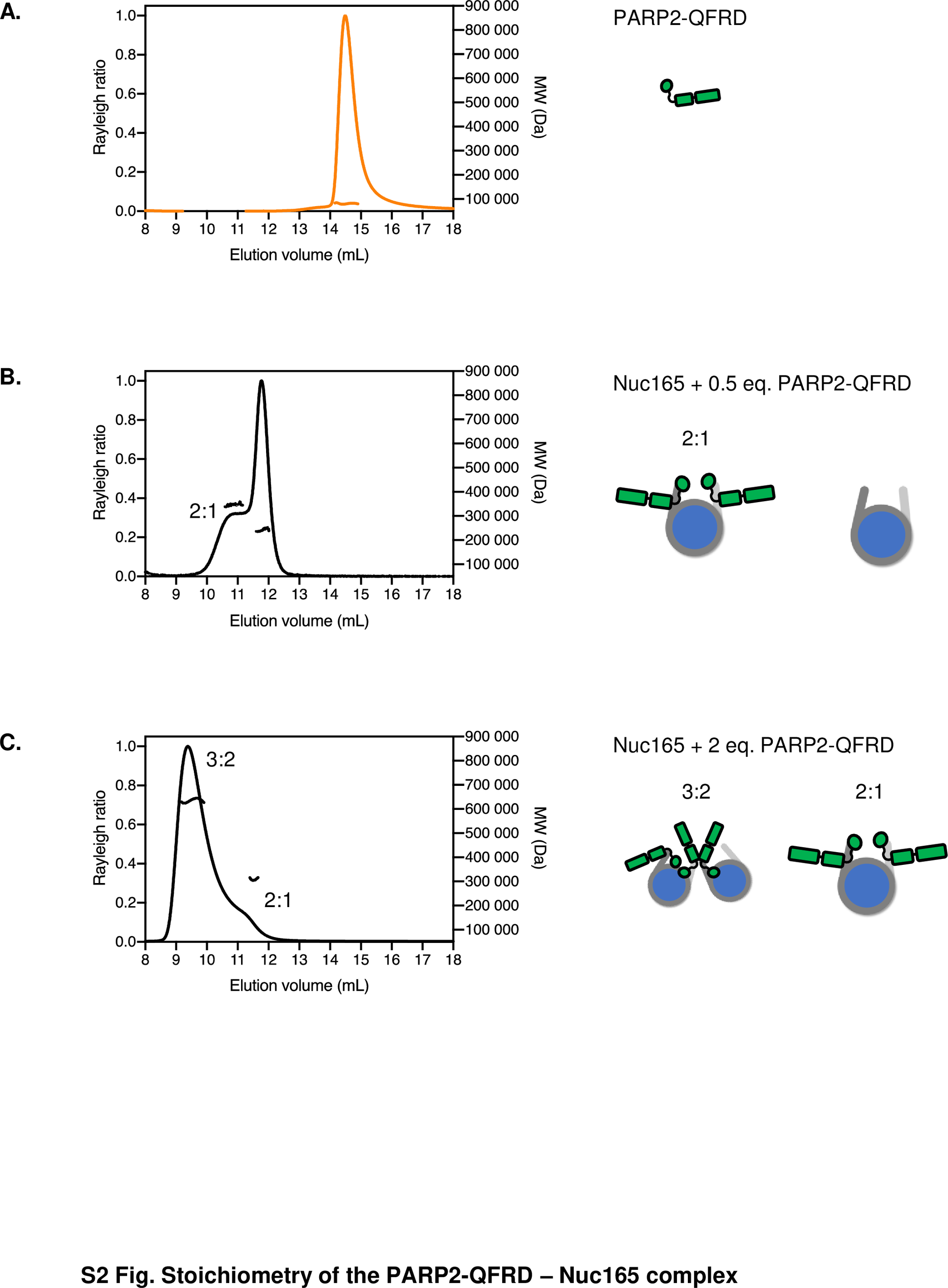

Supplement: S2 Fig — Size exclusion chromatograms and experimental molecular weights determined by SEC-MALS. All molecular weights are listed in Table 2. Stoichiometries consistent with the experimental molecular weights are depicted as cartoons. A: PARP2-QFRD. B: Nuc165 + 0.5 molar equivalent of PARP2-QFRD. C: Nuc165 + 2 molar equivalents of PARP2-QFRD. (TIF) [file pone.0240932.s002.tif]

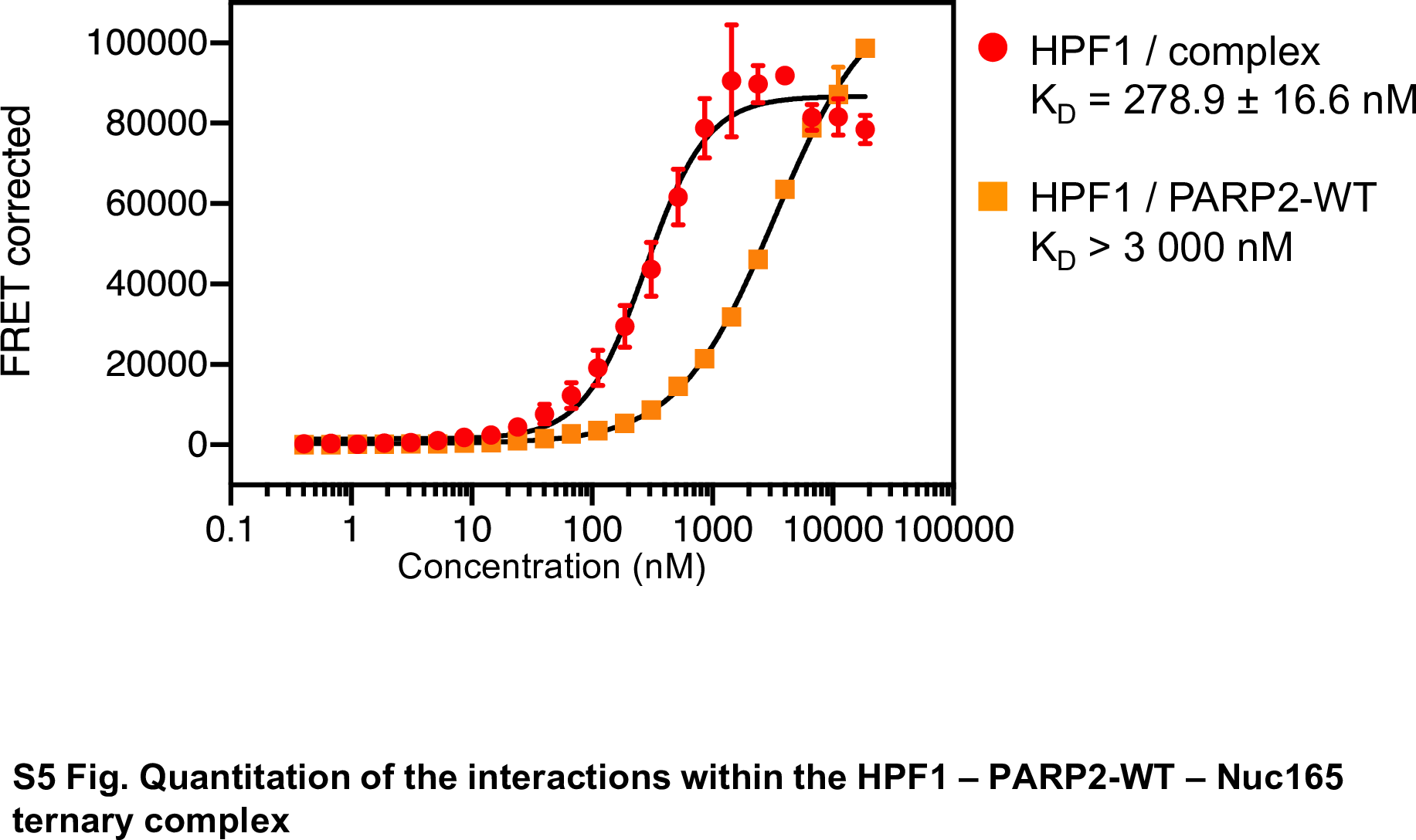

Supplement: S5 Fig — FRET binding curves of HPF1_A647 to the preformed PARP2-WT_A488•Nuc165 complex (1000 nM PARP2-WT, 100 nM Nuc165) and PARP2-WT_A488 (1000 nM). Points and error bars are the mean and standard deviation from three independent measurements (no visible error bar means that the error bar is smaller than the symbol used to plot the data point). Reported KD values are the mean and standard error of the mean. All KD values are listed in Table 3. (TIF) [file pone.0240932.s005.tif]

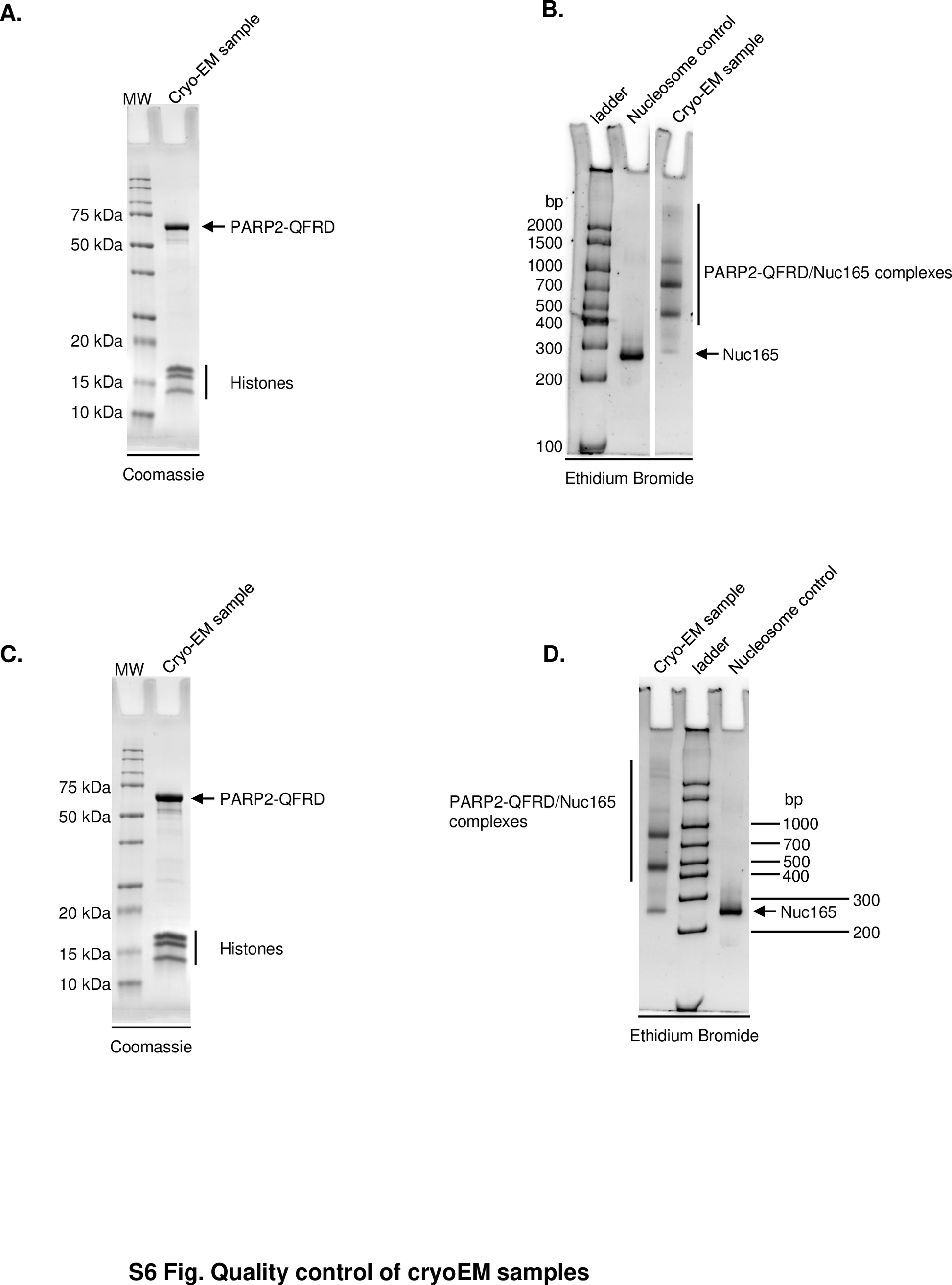

Supplement: S6 Fig — Samples were systematically analyzed by SDS-PAGE and 5% native PAGE after cryo-EM grid preparation. A: SDS-PAGE analysis of the sample that produced dataset 1. B: 5% native PAGE analysis of the sample that produced dataset 1. C: SDS-PAGE analysis of the sample that produced dataset 2. D: 5% native PAGE analysis of the sample that produced dataset 2. (TIF) [file pone.0240932.s006.tif]

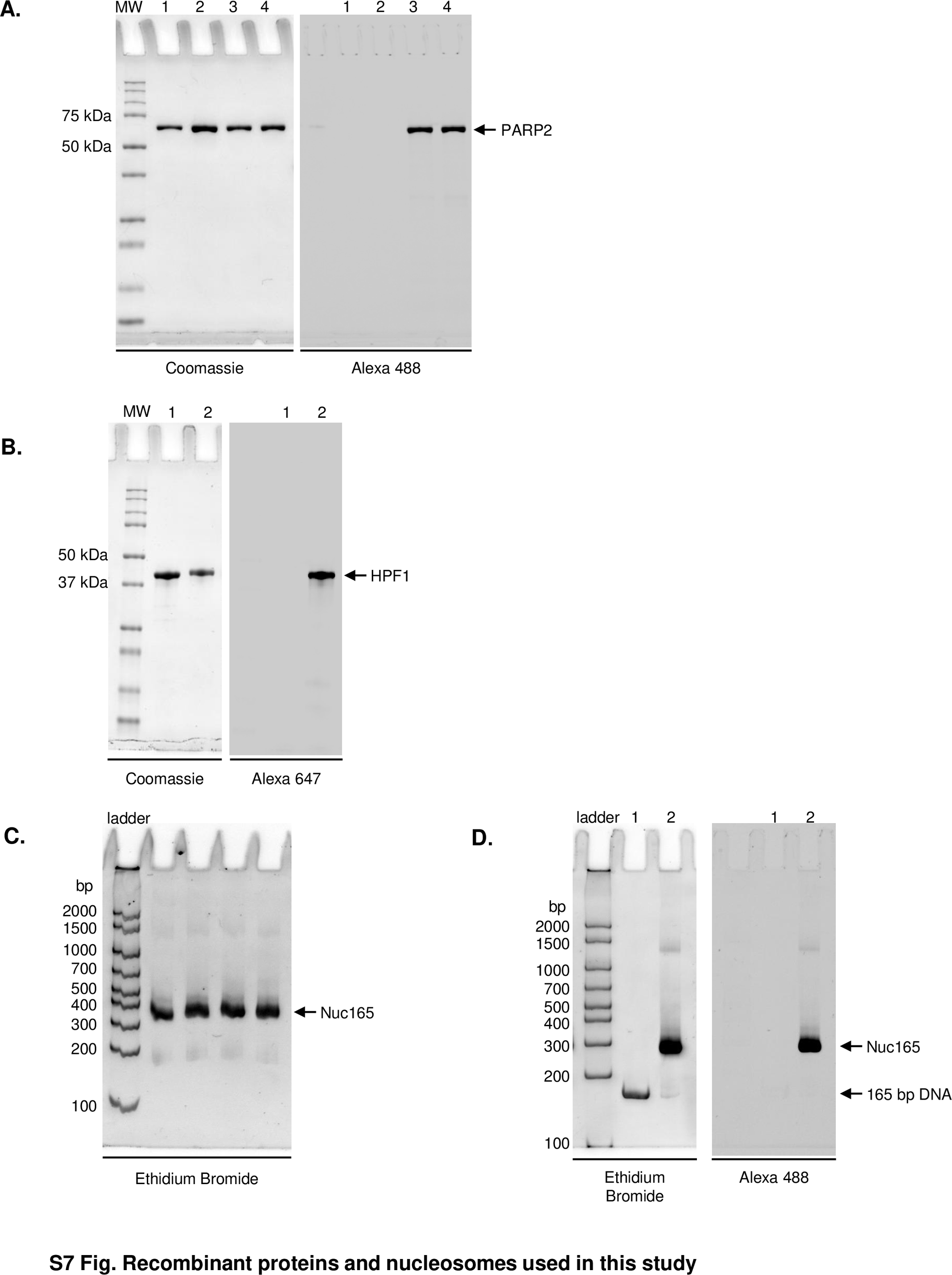

Supplement: S7 Fig — A: SDS-PAGE of 1 μg of purified recombinant PARP2-WT (lane 1), PARP2-QFRD (lane 2), Alexa488-labeled PARP2-WT (lane 3) and Alexa488-labeled PARP2-QFRD (lane 4). Proteins were detected by Coomassie staining (left panel) and covalent labeling was verified by Alexa488 fluorescence detection (right panel). B: SDS-PAGE of 1 μg of purified recombinant HPF1 (lane 1) and Alexa647-labeled HPF1 (lane 2). Proteins were detected by Coomassie staining (left panel) and covalent labeling was verified by Alexa647 fluorescence detection (right panel). C: 5% native PAGE of representative batches of Nuc165 used in this study (ethidium bromide staining). D: 5% native PAGE of Alexa488-labeled Nuc165 used in this study. The nucleosome was detected by ethidium bromide staining (left panel) and covalent labeling was verified by Alexa488 fluorescence detection (right panel). (TIF) [file pone.0240932.s007.tif]

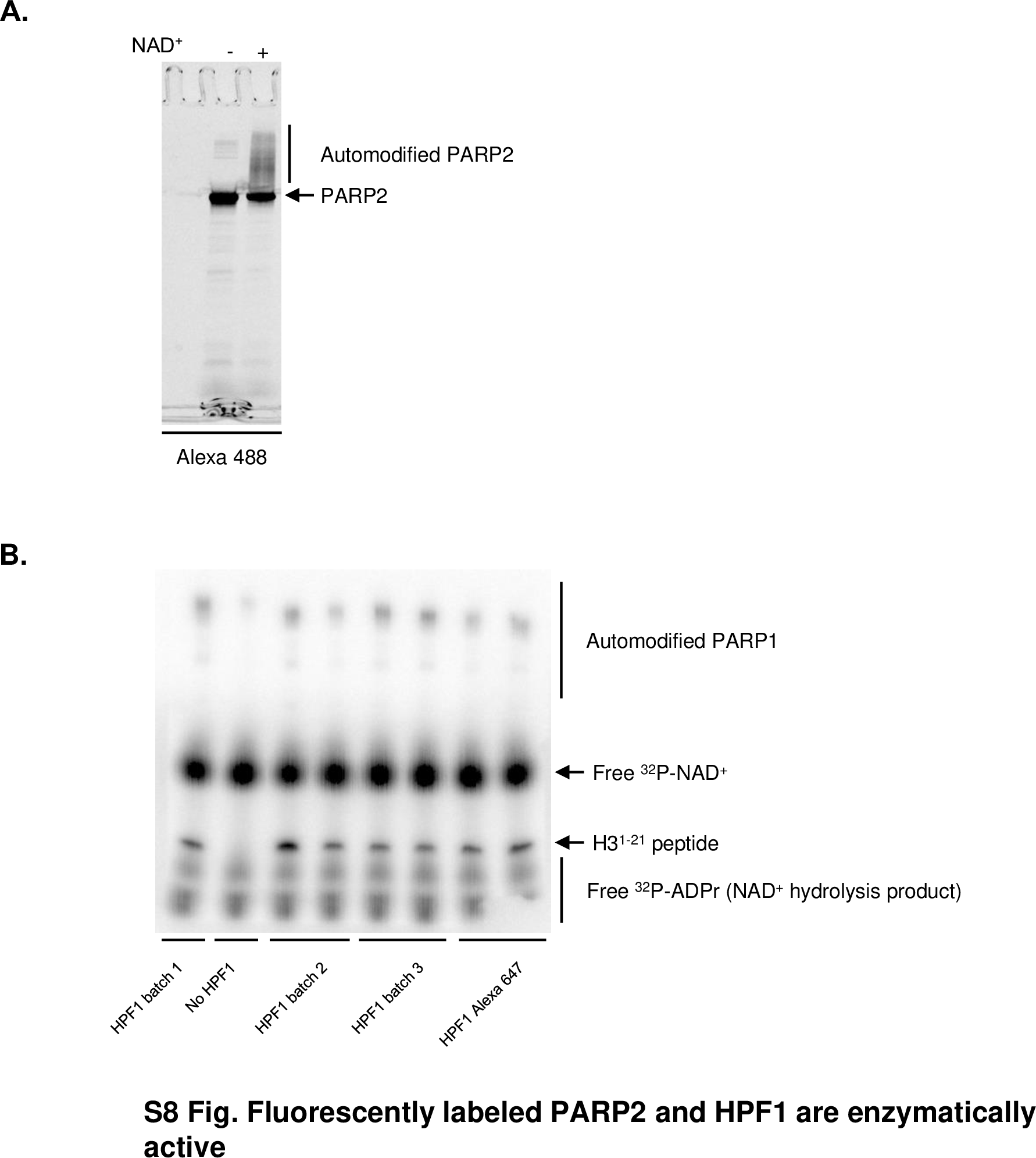

Supplement: S8 Fig — A: SDS-PAGE of unmodified and PARylated Alexa488-labeled PARP2-WT (Alexa488 fluorescence detection). B: Autoradiogram of an ADP-ribosylation reaction by PARP1 of a peptide of H31-21, in absence and presence of HPF1. Several representative batches of HPF1 were tested, as well as Alexa647-labeled HPF1. (TIF) [file pone.0240932.s008.tif]

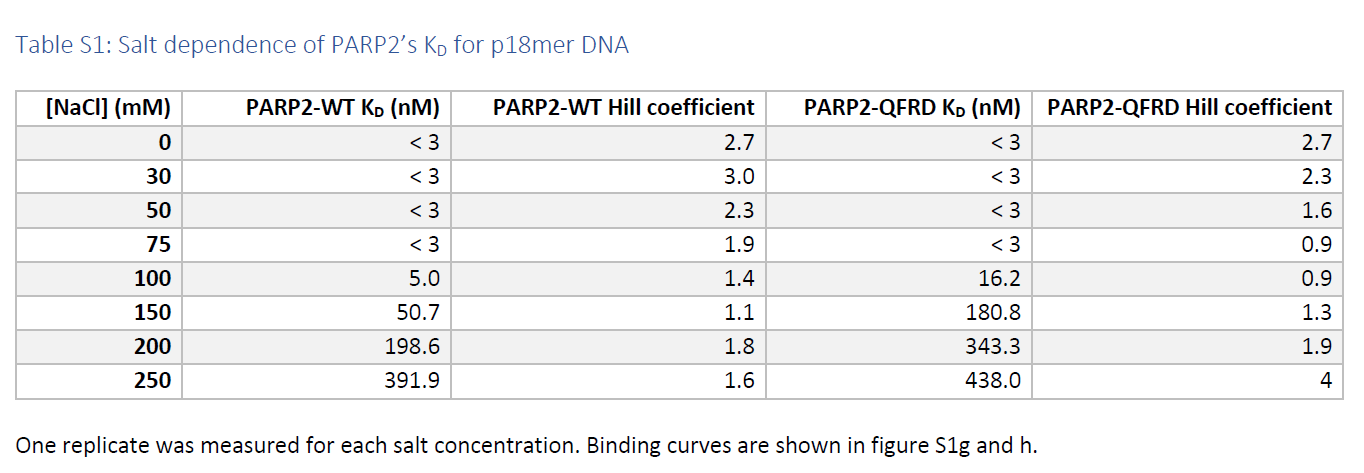

Supplement: S1 Table — (TIF) [file pone.0240932.s009.tif]

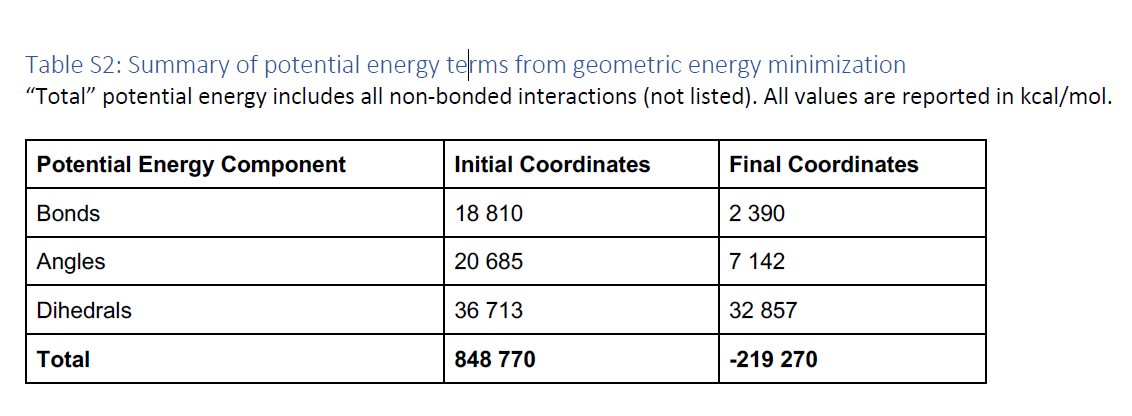

Supplement: S2 Table — (TIF) [file pone.0240932.s010.tif]
